# A Splicing Mutation in *Slc4a5* Results in Retinal Detachment and Retinal Pigment Epithelium Dysfunction

**DOI:** 10.3390/ijms23042220

**Published:** 2022-02-17

**Authors:** Gayle B. Collin, Lanying Shi, Minzhong Yu, Nurten Akturk, Jeremy R. Charette, Lillian F. Hyde, Sonia M. Weatherly, Martin F. Pera, Jürgen K. Naggert, Neal S. Peachey, Patsy M. Nishina, Mark P. Krebs

**Affiliations:** 1 The Jackson Laboratory, 600 Main Street, Bar Harbor, ME 04609, USA; gayle.collin@jax.org (G.B.C.); lanying_shi1234@163.com (L.S.); nurten.akturk@jax.org (N.A.); jeremy.charette@jax.org (J.R.C.); lillian.hyde@jax.org (L.F.H.); soniawxly@gmail.com (S.M.W.); martin.pera@jax.org (M.F.P.); juergen.naggert@jax.org (J.K.N.); 2 Department of Ophthalmic Research, Cole Eye Institute, Cleveland Clinic Foundation, 9500 Euclid Avenue, Cleveland, OH 44195, USA; yum@ccf.org (M.Y.); neal.peachey@va.gov (N.S.P.); 3 Department of Ophthalmology, Cleveland Clinic Lerner College of Medicine of Case Western Reserve University, 9500 Euclid Avenue, Cleveland, OH 44195, USA; 4 Research Service, Louis Stokes Cleveland VA Medical Center, 10701 East Boulevard, Cleveland, OH 44106, USA

**Keywords:** sensitized chemical mutagenesis screen, mouse genetics, inherited retinal disease, retinal dysplasia

## Abstract

Fluid and solute transporters of the retinal pigment epithelium (RPE) are core components of the outer blood–retinal barrier. Characterizing these transporters and their role in retinal homeostasis may provide insights into ocular function and disease. Here, we describe RPE defects in *tvrm77* mice, which exhibit hypopigmented patches in the central retina. Mapping and nucleotide sequencing of *tvrm77* mice revealed a disrupted 5’ splice donor sequence in *Slc4a5*, a sodium bicarbonate cotransporter gene. *Slc4a5* expression was reduced 19.7-fold in *tvrm77* RPE relative to controls, and alternative splice variants were detected. SLC4A5 was localized to the Golgi apparatus of cultured human RPE cells and in apical and basal membranes. Fundus imaging, optical coherence tomography, microscopy, and electroretinography (ERG) of *tvrm77* mice revealed retinal detachment, hypopigmented patches corresponding to neovascular lesions, and retinal folds. Detachment worsened and outer nuclear layer thickness decreased with age. ERG a- and b-wave response amplitudes were initially normal but declined in older mice. The direct current ERG fast oscillation and light peak were reduced in amplitude at all ages, whereas other RPE-associated responses were unaffected. These results link a new *Slc4a5* mutation to subretinal fluid accumulation and altered light-evoked RPE electrophysiological responses, suggesting that SLC4A5 functions at the outer blood–retinal barrier.

## 1. Introduction

The transport of nutrients, electrolytes, gases, water, and waste products between the retina and the systemic circulation is regulated by the blood–retinal barrier (BRB) [1]. Disruption of the BRB affects retinal homeostasis and visual function [2] and is implicated in major ocular diseases [3,4,5]. In most mammals, including humans and mice, the interface between the retina and circulation consists of two components: the inner BRB, which mediates transport between cells of the inner retina and the retinal vasculature, and the outer BRB, which mediates transport between the photoreceptor outer segments and the choroidal vascular bed (choriocapillaris). An essential component of the outer BRB is the retinal pigment epithelium (RPE), an epithelial monolayer that lies between the outer segments of the retina and the vascular endothelial cells of the choriocapillaris [1,6,7,8].

A core function of the RPE is to regulate subretinal fluid composition, which depends on the selective transport of ions and other solutes to and from this compartment, as well as on the transport of water to the choriocapillaris [1,6,7,8,9,10]. Ion, solute, and water transport are coordinated on short timescales to maintain an ionic environment suitable for photoreceptor signaling and recovery following light stimuli and on longer timescales to ensure a close apposition of outer segments with RPE apical processes, which is required for photoreceptor homeostasis. Physiological and pharmacological studies of vertebrate tissues and cultured cells have demonstrated that ATP-dependent transport of Na^+^ and K^+^ at the apical RPE provides the primary energetic basis for ion and water transport. Water transport is secondary to net ion efflux from the RPE, mainly of K^+^ and Cl^−^; other major solutes of the subretinal space that influence water efflux include H^+^, CO_2_, H_2_CO_3_, HCO_3_^−^, and lactate. Further, a number of RPE proteins are known or postulated to mediate solute and water transport, such as the apical Na^+^, K^+^ ATPase, potassium channel KCNJ13 (Kir7.1), Na^+^-K^+^-2Cl^−^ cotransporter, and Na^+^-H^+^ antiporter; basolateral chloride channels CFTR, BEST1, and CLCN2 (ClC-2) and the Cl^−^-HCO_3_^−^ exchanger SCL4A2 (AE2); apical and basolateral AQP1 (aquaporin), monocarboxylate transporters MCT1 and MCT3, and sodium bicarbonate cotransporters; and intracellular carbonic anhydrase, which converts dissolved CO_2_ gas to H_2_CO_3_ [1,6,7,8,9,10]. However, uncertainties remain about the contribution of these specific transporters and channels to RPE ion, solute, and water transport in vivo, which may benefit from studying animal models that disrupt them. For example, the retinal degenerative phenotype of a *Clcn2* knockout mouse was rescued by an RPE-specific *Clcn2* expression construct, demonstrating that CLCN2 function in the RPE is critical for retinal homeostasis. Surprisingly, the expressed protein was localized to the apical RPE in vivo, raising the possibility that it does not function as a basolateral transporter as widely postulated [11].

In the present work, we characterize a mutant mouse line, *tvrm77,* identified by the Translational Vision Research Models (TVRM) program at the Jackson Laboratory (JAX). In this program, new mouse models of ocular disease are developed from C57BL/6J (B6) mice and B6-derived mutant strains by chemical mutagenesis and phenotypic screening [12,13]. As last tabulated in 2016, 34 disease models have been identified through the TVRM program and are currently available for distribution to the scientific community, while additional heritable mutants, including *tvrm77*, remain to be characterized [14]. In an earlier summary of the program, the *tvrm77* phenotype was described as having central retinal patches due to autosomal recessive inheritance of a mutation on chromosome (Chr) 6 [12].

Here, we show that the *tvrm77* phenotype is associated with a mutation in *Slc4a5*, which encodes an integral membrane solute carrier of the bicarbonate transporter family [15]. *Slc4a5* is widely transcribed in mammalian tissues [16], and SLC4A5 (also known as NBC4 or NBCe2) isoforms arising from alternative promotors and splicing are detected at the plasma membrane of epithelial cells in the bile duct and liver [17], the kidney outer medullary collecting duct [18], and the brain choroid plexus [19,20,21,22,23]. Physiological studies including the analysis of *Slc4a5* knockout mice established that SLC4A5 functions in these tissues as an electrogenic sodium bicarbonate cotransporter [20,21,23,24,25,26]. Previous analysis of ocular pathology in an *Slc4a5* knockout strain revealed retinal detachment and a loss of retinal function, which were attributed to the loss of photoreceptor and retinal ganglion cells [21]. *SLC4A5* mutations have not been linked to any monogenic human diseases, although variants have been associated with an increased risk of elevated blood pressure and an increased sensitivity of blood pressure to a high sodium intake [27,28,29,30].

Given that SLC4A5 transport functions have been documented in epithelial tissues [18,19,20,21,22,23] and that retinal detachment in mice is associated with RPE dysfunction [31,32,33], we considered the hypothesis that a loss of SLC4A5 function in the RPE might contribute to the reported retinal detachment and accompanying ocular phenotypes in *Slc4a5* mutant mice. Our studies support this hypothesis and advance our understanding of SLC4A5 function at the outer BRB.

## 2. Results

### 2.1. Identification of a New Slc4a5 Allele

**Figure 1:**
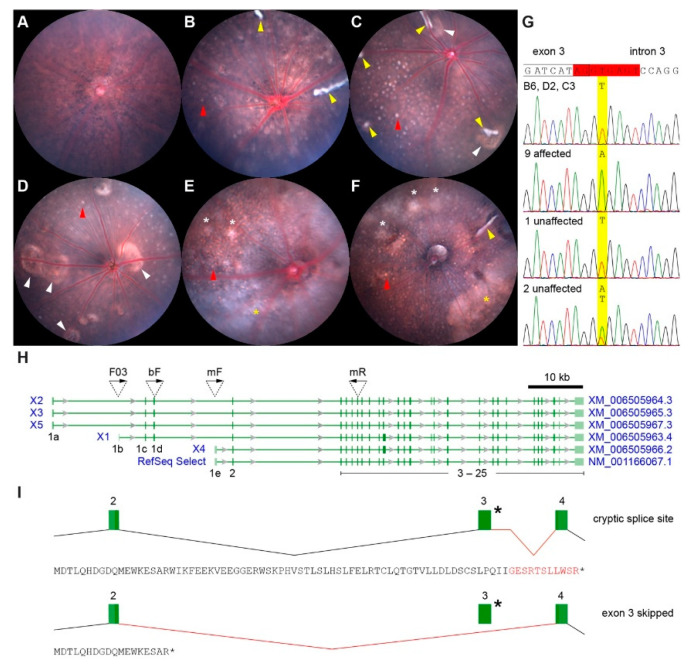
Representative fundus phenotypes of *tvrm77* mice and identification of the causative mutation. (**A**) Bright-field fundus image of a female (**F**) B6 mouse at 1 month of age. (**B**–**F**) Fundus images of *tvrm77* mice (male (M), 0.9; F, 2.1; M, 1.7; M, 5.0; and M, 11.5 months of age). Variable phenotypes were noted, including hyperreflective white lines (yellow arrowheads), hypopigmented patches (white arrowheads), and, in older mice (**E**,**F**), diffuse spots (white asterisks) and a white haziness or halo toward the peripheral retina (yellow asterisks). Small bright spots were evident, particularly in older *tvrm77* eyes, throughout the region imaged (red arrowheads indicate a single spot as an example). (**G**) DNA sequence of the splice donor exon 3–intron 3 junction of the mouse *Slc4a5* gene (RefSeq Select mRNA transcript NM_001166067.1 is used as the reference for exon and intron numbering). DNA bases corresponding to the conserved mRNA 5’ splice donor sequence of exon 3 are highlighted (red box), and the nucleotide affected in *tvrm77* is highlighted in yellow. *Slc4a5* DNA sequencing chromatograms of the parental strains (B6, C57BL/6J; D2, DBA/2J; C3, C3A.BLiA-*Pde6b*^+^/J) and mice from the *tvrm77* maintenance colony, as well as mapping crosses, are shown. The number of mice sequenced with the observed phenotype is indicated. (**H**) Protein-coding transcripts for mouse *Slc4a5* on Chr 6 listed at the National Center for Biotechnology Information (Reference GRCm39 C57BL/6J, NC_000072.7 (8316661183293218)). Transcripts include one RefSeq Select and five variant transcripts (X1–X5; transcripts and corresponding accession sequences are labeled in blue). Exons are numbered as in the RefSeq Select transcript, except that the first and upstream exons in transcript variants are numbered 1a–1e to retain identical numbering for downstream exons common to all transcripts. The locations of exon-specific sequencing primers for cDNA analysis are indicated by inverted triangles with arrows indicating 5′–3′ orientation. Coding exons, dark green; non-coding exons, light green. (**I**) Alternatively spliced transcripts as assessed by cDNA analysis of homozygous *tvrm77* RPE. Normal (black lines) and alternative (red lines) *Slc4a5* mRNA splicing events are shown; exons are numbered as in (**A**). The mutation in intron 3 is indicated by an asterisk. Transcripts using a cryptic splice site in intron 3 or skipping exon 3 entirely were observed. The SLC4A5 polypeptide predicted from the RefSeq Select transcript in *tvrm77* mice is indicated below each splicing diagram. The first position corresponds to the initiating methionine; amino acids absent from the wild-type sequence are indicated in red.

To extend the description of the “central patches” phenotype we reported previously [12], indirect ophthalmoscopy and bright-field fundus imaging with processing in Fiji [34] was performed on presumed homozygous *tvrm77* mice, which had been backcrossed with B6 mice for five generations (N_5_) and intercrossed. The colony was maintained by mating phenotypically affected individuals. Once the causative mutation was identified (see below), mice founded from homozygous progeny of an additional backcross (N_6_) were also analyzed. Compared to B6 controls (Figure 1A), the eyes of *tvrm77* mice exhibited multiple abnormalities, including hypopigmented central patches (white arrowheads, Figure 1C,D), which were sometimes associated with “white-line” lesions (yellow arrowheads, Figure 1B,C,E); numerous small spots distributed across the retina (red arrowheads, Figure 1B–F); and a cloudy white halo or haziness toward the periphery (yellow asterisks, Figure 1E,F). B6 × DBA/2J and B6 × C3A.BLiA-*Pde6b*^+^/J mapping crosses based on this aggregate phenotype localized the *tvrm77* mutation to a 12 Mbp region of Chr 6. Targeted sequencing of cDNA prepared from whole eye mRNA revealed a deletion of *Slc4a5* exon 3 in *tvrm77* samples (exon numbering based on RefSeq Select sequence NM_001166067.1). Further sequencing of *Slc4a5* genomic DNA identified a T-to-A transversion in intron 3 of *tvrm77* mice, likely disrupting a 5’ splice donor sequence (Figure 1G, red box). To confirm the association of this mutation with the mutant phenotype, the *Slc4a5* gene was sequenced in five affected mice from the presumed homozygous colony, four affected mice from the mapping intercross, and three unaffected intercross progeny (Figure 1G). A statistically significant association of the homozygous mutation with the affected phenotype was observed (*p* = 4.5 × 10^−3^; two-tailed Fisher’s exact test). The full designation for this model is C57BL/6J*^-^Slc4a5^tvrm77^*/Pjn; for simplicity, we retain the original strain designation *tvrm77* for the remainder of the paper, and unless otherwise indicated, the genotype is homozygous for the mutant allele.

### 2.2. Analysis of RPE Slc4a5 mRNA Transcripts

The ocular phenotypes of *tvrm77* mice suggested that the gene is expressed in the posterior eye. Previous cell-specific transcriptome analysis of the mouse retina indicated that *Slc4a5* mRNA was enriched 3.4- to 3.9-fold on average in horizontal cells relative to other retinal cell types [35,36]. This biased expression of the mRNA in horizontal cells is consistent with the immunolocalization of SLC4A5 predominantly to the outer plexiform layer [21], which contains horizontal cell processes. However, murine *Slc4a5* and its mammalian orthologs are also expressed in epithelia of many tissues [17,18,19,20,21,22,23]. Notably, *Slc4a5* mRNA has been detected in the mouse iris, ciliary bodies, and RPE at levels that exceed retinal expression 15-, 35-, and 76-fold, respectively (BioGPS, GeneAtlas MOE430, gcrma). *Slc4a5* transcripts have also been detected in the RPE of C57BL/6NCrl and C57BL/6J mice by bulk and single-cell RNA sequencing, respectively [37,38].

To assess the effects of the *tvrm77* mutation on *Slc4a5* expression in the RPE, mRNA levels in RPE-enriched cell lysates were determined by qRT-PCR. *Slc4a5* mRNA was detected in B6 samples and was decreased 19.7-fold in *tvrm77* samples (*p* = 0.05, two-tailed Student’s t-test; n = 4 and 3 for B6 and *tvrm77*, respectively).

Multiple promoters and alternative splicing events generating six transcripts are documented for the murine *Slc4a5* gene (Figure 1H, RefSeq Select and variant transcripts X1–X5). PCR products amplified from cDNA prepared from B6 and *tvrm77* RPE RNA samples were sequenced to determine the effect of the mutation on transcript processing. The amplification of B6 RPE samples using primers mF and mR (see Data S1 for primer sequences) yielded a product matching the known sequences of the RefSeq Select and X4 transcripts, which are identical within the region amplified. The use of primers F03 and mR identified products with a sequence corresponding to that of X1, indicating that transcripts expressed from an upstream promoter previously identified in rats [22] are also present in the wild-type mouse RPE. By contrast, analysis of *tvrm77* RPE with the mF and mR primers revealed two novel splicing events. In one, the use of a cryptic splice site within intron 3 yielded a splice variant that includes a portion of the downstream intron linked to exon 4 (Figure 1I, cryptic splice site). The resulting transcript is predicted to encode a short N-terminal polypeptide that includes 11 non-native amino acids prior to premature termination. In the other novel splicing event, exon 3 is skipped entirely, again leading to premature termination of the polypeptide (Figure 1I, exon 3 skipped). Use of the cryptic splice site was also indicated in products corresponding to the X1 transcript, but insufficient product was available to verify exon 3 skipping. Together with the qRT-PCR results, these findings indicate that known *Slc4a5* transcripts are expressed in the mouse RPE and altered by the *tvrm77* mutation.

Independent of promoter selection, if translated, the truncated SLC4A5 polypeptide fragments encoded by *tvrm77* splice variants are predicted to lie within the cytoplasmic N-terminal domain. These fragments would not retain the normal sodium bicarbonate transport function of SLC4A5, which is predicted to map to the transmembrane domain based on structural studies of the closely related transporter SLC4A4 [39]. Premature termination and subsequent nonsense-mediated RNA decay may account for the lower transcript accumulation observed in *tvrm77* mice compared to B6 controls.

### 2.3. SLC4A5 Cellular Localization in Human RPE Cells

**Figure 2:**
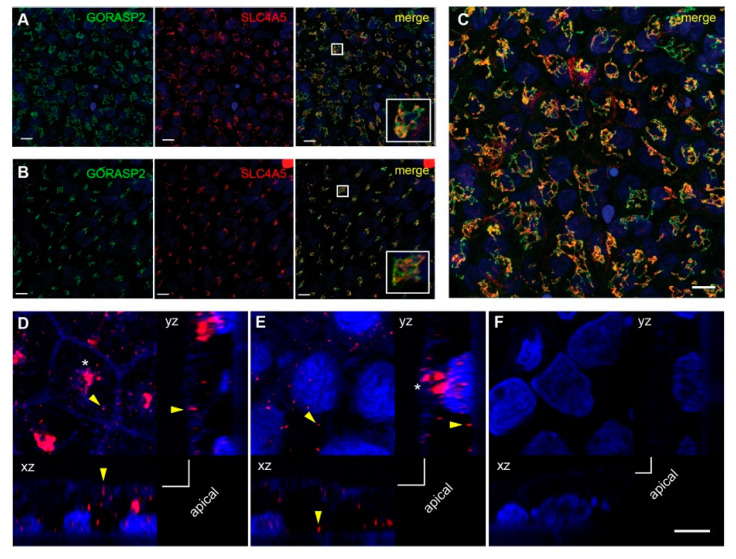
Expression of SLC4A5 in cultured human RPE differentiated from pluripotent stem cells. (**A**,**B**) Double-label indirect immunofluorescence micrographs of staining for Golgi marker GORASP2 (green) and SLC4A5 (red) on RPE differentiated (Day 30) from human embryonic stem cell line WA09 (**A**) or human iPSC line A04 (**B**) and grown on tissue culture plastic. Nuclei are counterstained with DAPI (blue). Scale bar, 10 µm. (**C**) Higher power magnification of double-label staining of WA09 cells as in (**A**,**B**). Scale bar, 10 µm. (**D**,**E**). Possible membrane distribution of SLC4A5, as determined by deconvolution confocal microscopy, in WA09 cells grown on transwell membranes and stained with anti-SLC4A5 antibody and DAPI. Single z-slices of the same image stack are shown adjacent to the corresponding orthogonal projections (xz, yz). A falloff of faint blue cellular fluorescence was used to approximate the apical cell boundary (corner brackets); the transwell membrane surface defined the basal cell boundary. Bright SLC4A5 puncta (yellow arrowheads) near the apical (**D**) and basal (**E**) boundaries were distinct from predominant staining in the central cell (asterisk) and may therefore represent membrane-associated rather than Golgi-localized SLC4A5. (**F**) A single z-slice and orthogonal projections of an image stack acquired from the same cells, which were treated identically except for the omission of primary antibody. SLC4A5 signal was not observed. Scale bar in (**F**), 5 µm, applies to (**D**–**F**).

We were unsuccessful using commercially available SLC4A5 antibodies for immunofluorescence microscopy of mouse ocular tissue. Therefore, to determine whether the SLC4A5 protein was expressed in RPE cells, we examined two lines of pluripotent stem cells (WA09 human embryonic stem cell, hESC; A04, human induced pluripotent stem cell, hiPSC) that were differentiated into RPE using an established protocol [40,41]. In RPE from either stem cell line grown on tissue culture plastic, SLC4A5 immunostaining was readily evident in all cells, displaying a pattern consistent with localization to the Golgi apparatus (Figure 2A–C). This subcellular localization was confirmed by double-label staining with an antibody against the Golgi marker GORASP2 (Figure 2A–C). Further analysis of WA09 cells by deconvolution microscopy revealed SLC4A5 puncta near both the apical and basal plasma cell boundaries (Figure 2D,E) and at intracellular locations that may represent protein associated with secretory trafficking components. SLC4A5 staining was not observed among control samples, in which primary SLC4A5 antibody was omitted (Figure 2F). In summary, *Slc4a5* mRNA and SLC4A5 protein are expressed in mouse RPE and cultured human RPE cells, respectively. Based on this evidence and our RNA analysis, a substantial decrease in SLC4A5 levels and function is expected in the RPE of *tvrm77* mice.

### 2.4. Change in Lesion Prevalence with Age and Generation

Evidence that the *Slc4a5* gene and protein are normally expressed in the RPE led us to consider whether RPE morphology and function were altered in *tvrm77* mice. We first reviewed the prevalence of indirect ophthalmoscopy phenotypes with age to assess disease onset and progression. White-line lesions in one or both eyes were observed in 65% of *tvrm77* mice (28/43 mice, including 24 F and 19 M mice) at 0.9–1.2 months of age, the earliest ages examined. The prevalence of these lesions was similar at older ages: 72% (26/36 mice, 17 F and 19 M) at 1.7–3.7 months and 62% (18/29, 19 F and 10 M) at 4.1–11.5 months of age. Thus, this phenotype is established early and remains stable (Figure S1). By contrast, the prevalence of a peripheral halo or haziness increased from 14% (6/43) at 0.9–1.2 months of age to 97% (35/36) and 100% (29/29) in the two older groups, respectively, indicating progression of this phenotype with age (Figure S1). These results suggested that white-line lesions could be characterized in young adult mice, whereas the peripheral halo phenotype needs to be examined in older mice.

Indirect ophthalmoscopy also indicated that the white-line lesion phenotype decreased in frequency with an additional backcross to B6 from N_5_ to N_6_. At 0.9–1.2 months of age, a statistically significant decrease in white-line lesion prevalence in one or both eyes was observed among mice from the later generation (N_5_: 93%; 14/15, 6 F and 9 M; N_6_: 50%; 14/28, 18 F and 10 M; chi-square test, *p* = 4.4 × 10^−3^). This observation raises the possibility that, although the phenotype appears to require the *tvrm77* mutation, the pathogenesis of the white-line lesion phenotype may be influenced by additional mutations in as-yet-unidentified modifier genes.

### 2.5. Noninvasive Imaging of White-Line Lesions and Hypopigmented Patches

**Figure 3:**
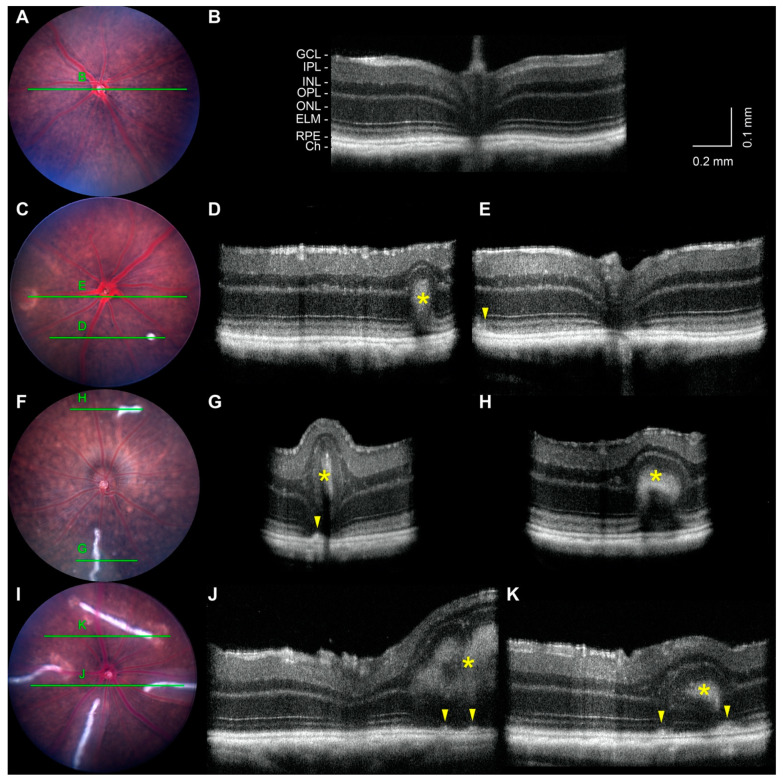
Non-invasive imaging of white-line lesions at 1 month of age. (**A**) Bright-field fundus image of a B6 eye. (**B**) B-scan taken from an OCT volume dataset of the same eye. The full OCT dataset is presented in Video S1. GCL, ganglion cell layer; IPL, inner plexiform layer; INL, inner nuclear layer; OPL, outer plexiform layer; ONL, outer nuclear layer; RPE, retinal pigmented epithelium; Ch, choroid. (**C**,**F**,**I**) Fundus images of *tvrm77* eyes showing white-line lesions of different sizes. Green lines mark the locations of the corresponding OCT B-scans. (**D**,**E**,**G**,**H**,**J**,**K**) B-scans taken from OCT volume datasets of the same eyes. The full OCT volume datasets are presented in Videos S2–S4. Lesion hyperreflective cores (asterisks) and thickening of the RPE (arrowheads) are indicated. Fundus images were scaled and aligned with the corresponding *en face* OCT datasets using Fiji. The vertical dimension of the OCT images was increased two-fold to enhance image detail. Scale bar in (**B**) applies to (**D**,**E**,**G**,**H**,**J**,**K**).

Noninvasive imaging was performed to characterize the posterior ocular defects. In agreement with indirect ophthalmoscopy, fundus imaging of B6 (Figure 3A) and *tvrm77* mice (Figure 3C,F,I) at 0.9–1.0 months of age confirmed the absence of white-line lesions in control eyes (0%; 0/11 eyes from 4 F and 7 M mice) and their presence in *tvrm77* eyes (48%; 20/42 eyes, 12 F and 16 M mice). The association of lesions in fundus images of one or both eyes (12/28) with the *tvrm77* genotype was statistically significant (chi-square test, *p* = 9.1 × 10^−3^). Lesions ranged in number from one to five among fundus images of 20 affected eyes of 6 F and 10 M mice and varied in length from small dots to lines occupying more than a third of the diameter of the fundus image (Figure 3C,F,I). Hypopigmented patches were absent in fundus images of B6 control mice (0/11, 4 F and 7 M mice) but present in 64% of images of *tvrm77* eyes (27/42, 12 F and 16 M mice). The association of hypopigmented patches in one or both eyes (19/28) with the *tvrm77* genotype was also statistically significant (chi-square test, *p* = 1.4 × 10^−4^). These lesions typically feature a bright border surrounding darker islands (Figure 3C,I; Figure 1C,D), typical of focal RPE changes at neovascular lesions, as characterized by fundus imaging in other mouse models [42,43,44]. Hypopigmented patches were often observed near white-line lesions (Figure 3I).

OCT was also performed on the eyes of B6 and *tvrm77* mice at 0.9–1.0 months of age, including some of the same eyes assessed by bright-field fundus imaging (Figure 3). Eyes of B6 mice exhibited a normal fundus appearance (Figure 3A) and normal retinal lamination by OCT (Figure 3B, Video S1). Among affected *tvrm77* mice, the shortest dot-like white-line lesions (Figure 3C) appeared as a hyperreflective intrusion into the outer retina, with minimal effect on total retinal thickness (Figure 3D, asterisk; Video S2). A thinned outer nuclear layer was detected above the hyperreflective region, and the outer plexiform and inner nuclear layers (OPL and INL, respectively) were distorted above it. A hyporeflective gap was observed at the base of the lesion. Focal RPE thickening was observed near hypopigmented patches, consistent with the OCT appearance of RPE alterations known to accompany retinal neovascular structures [45,46,47]. In longer white-line lesions (Figure 3F,I), a hyperreflective core was observed by OCT (Figure 3G,H,J,K, asterisks; Videos S3 and S4), but the distortion of retinal layers and local increase in total retinal thickness was more pronounced than that associated with dot-like lesions. Focal thickening of the RPE layer was observed in the largest lesions (Figure 3H,J,K, arrowheads), often occurring in the same region as the hypopigmented lesions detected by brightfield fundus imaging (compare Figure 3I,K). In the largest lesions, a thinned ONL appeared both below and above the hyperreflective core along much of its length (Figure 3G,H,J,K, asterisks; Videos S3 and S4), although at some locations, the ONL was interrupted and the hyperreflective core extended from the OPL toward the outer retina (Figure 3K).

We searched the literature for retinal lesions resembling the white-line lesions, particularly as characterized by OCT. Outer and full-thickness retinal folds that develop as a complication of rhegmatogenous retinal detachment surgery [48] appear as pale linear features by color fundus imaging and corresponding hyperreflective intrusions by OCT, which distend the overlying retinal layers and cause a local increase in total retinal thickness [49,50,51]. Retinal folds with similar color fundus imaging and OCT features are also found in inherited forms of canine retinal dysplasia [52]. Our phenotypic observations suggest that the white-line lesions in *tvrm77* mice are retinal folds.

### 2.6. Retinal Detachment and Layer Thinning

**Figure 4:**
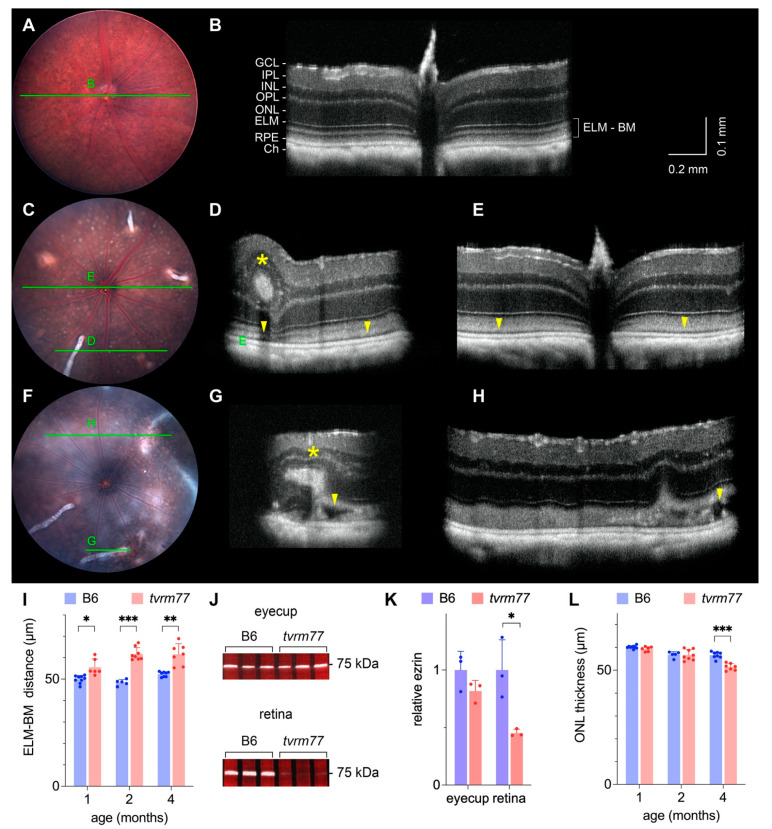
Non-invasive imaging of white-line lesions at 2 and 4 months of age. (**A**) Bright-field fundus image of a B6 eye at 4 months of age. (**B**) B-scan taken from an OCT volume dataset of the same eye. The full OCT dataset is presented in Video S5. Layers are labeled as in Figure 3. (**C**) Fundus images of *tvrm77* eye at 2 months of age. Green lines mark the locations of the corresponding OCT B-scans. (**D**,**E**) B-scans taken from an OCT volume dataset of the same eye. Hyporeflective regions (arrowheads) indicating subretinal fluid between the RPE and retina are evident below white-line lesions (asterisk) and in regions where lesions are not apparent. The full OCT volume dataset is presented in Video S6. (**F**) Fundus images of *tvrm77* eye at 4 months of age. Overall haziness of the image is more apparent than at 2 months of age. (**G**,**H**) B-scans of the same eye indicate more pronounced hyporeflective regions (arrowheads) both below white-line lesions (asterisk) and in regions where lesions are not apparent. The full OCT volume dataset is presented in Video S7. Fundus images were aligned to the corresponding OCT datasets using Fiji. The vertical scale of the OCT images was increased two-fold to enhance image detail. (**I**) ELM-BM distance as a function of age as measured by OCT in B6 and *tvrm77* eyes. (**J**) Western analysis for ezrin transfer assay. Lysates of total eyecup or peeled retina were analyzed by Western blotting using antibody against ezrin. Blot images are pseudocolored to indicate Ponceau S protein (red) and ezrin fluorescence (grayscale). The position of a 75 kDa molecular weight protein standard is indicated. (**K**) Quantitation of the relative ezrin levels in eyecup and peeled retina from the Western blot shown in (**J**). Relative ezrin values were normalized by dividing by the mean B6 value. Full-length blots are provided in Figure S2. (**L**) ONL thickness as a function of age as measured by OCT in B6 and *tvrm77* eyes. In (**I**,**K**,**L)**, bars indicate mean (±SD), and statistical significance is indicated by asterisks: * *p* < 0.01; ** *p* < 0.001; *** *p* < 0.00001. Scale bar in (**B**) applies to (**D**,**E**,**G**,**H**).

We hypothesized that the peripheral haziness or halo observed by indirect ophthalmoscopy in older *tvrm77* mice corresponds to regions of retinal detachment, as documented in fundus images obtained from retinal detachment models, such as homozygous *Prkcq^rpea1^* mice [32]. Fundus images centered on the optic nerve of B6 mice did not indicate haziness at 1, 2, or 4 months of age (Figure 3A, Figure 4A,B) or in *tvrm77* mice at 1 or 2 months of age (Figure 3C, Figure 4C). In B6 mice, the corresponding OCT volume datasets indicated a close apposition of the retina with the RPE. By contrast, in *tvrm77* mice at 4 months of age or older, substantial haziness was apparent within the imaged region (Figure 4D). At this age, OCT indicated disorganization of the outermost retinal layers and a variable hyporeflective region between the retina and RPE, which was more pronounced toward the periphery (Figure 4G,H, arrowheads; Videos S6 and S7). Hyporeflective regions were apparent even at 1 and 2 months of age (Figure 3G,H, Figure 4D,E, arrowheads; Videos S2–S4). In OCT scans of human patients and animal models, hyporeflective areas between the retina and RPE indicate subretinal fluid, associated with pathological [53,54], post-surgical [55], or experimentally induced retinal detachment [56].

To examine the progression of the retinal detachment phenotype, retinal layer thicknesses in OCT volume datasets were measured as a function of age. The distance between the external limiting membrane (ELM) and the scleral surface of the RPE, which we denote as the ELM–Bruch’s membrane distance (ELM-BM), showed a statistically significant increase in *tvrm77* compared to B6 control mice at all ages examined (Figure 4I, two-tailed Student’s t-test; *p* = 1.8 × 10^−3^; *p* = 1.5 × 10^−6^; and *p* = 1.9 × 10^−4^ at 1, 2, and 4 months of age, respectively; B6, n = 5–9; *tvrm77*, n = 5–8). These results suggest that retinal detachment initiates during early development in *tvrm77* mice.

To provide additional evidence for retinal detachment, we used a retina–RPE adhesion assay that examines the transfer of ezrin from the RPE apical surface to the retina, which is measured by Western analysis after the retina is peeled from the RPE [32,57]. A statistically significant decrease in the relative amount of ezrin detected in peeled retinas was observed in *tvrm77* compared to B6 eyes (Figure 4J,K; two-tailed Student’s t-test, *p* = 0.023, n = 3 for both strains). The total amount of ezrin in *tvrm77* and B6 eyecups was similar, suggesting that ezrin accumulation was unaffected by the mutation. These results indicate a decreased association of the retina and RPE consistent with retinal detachment.

Retinal detachment is often associated with progressive degeneration of the photoreceptor layer [58]. To test for potential degeneration, we measured the ONL thickness by OCT at 1, 2, and 4 months of age (Figure 4L). ONL thickness showed a statistically significant decrease in *tvrm77* mice compared to B6 mice at 4 months of age (two-tailed Student’s t-test, *p* = 1.2 × 10^−5^; B6, n = 8; *tvrm77*, n = 7). Thus, the *tvrm77* mutation is also associated with retinal degeneration characterized by a loss of photoreceptor cells.

### 2.7. Histological Characterization of Retinal Folds and Neovascular Lesions

**Figure 5:**
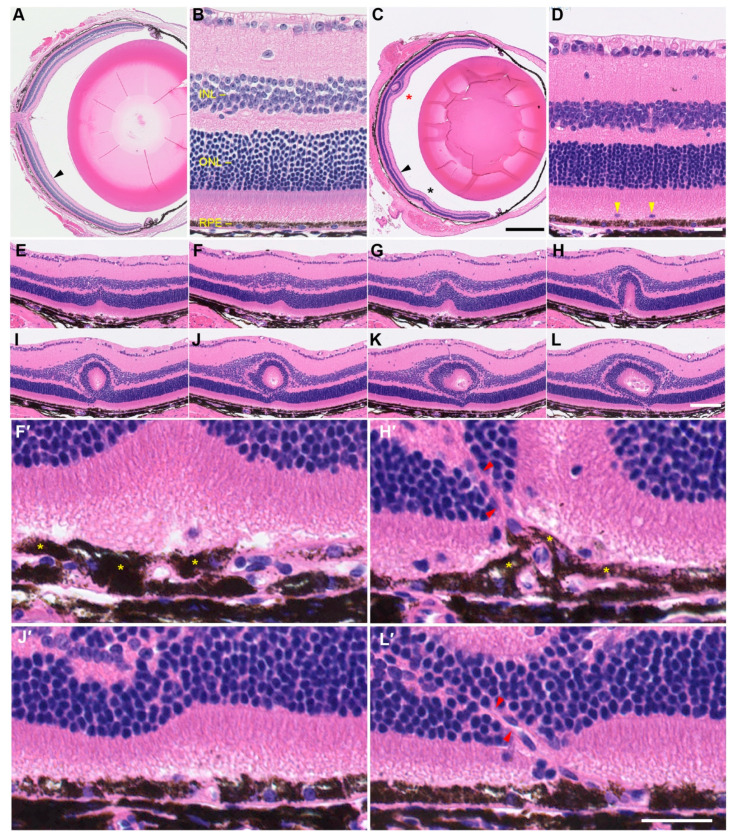
Hematoxylin- and eosin-stained ocular sections of control (n = 4) and *tvrm77* mice (n = 4) at 2 months of age. (**A**) B6 eye. (**B**) Detail of (**A**), arrowhead, indicating normal lamination. GCL, ganglion cell layer; INL, inner nuclear layer; ONL, outer nuclear layer; RPE, retinal pigment epithelium. (**C**) *tvrm77* eye. Retinal folds, asterisks. Scale bar, 500 µm, also applies to panel (**A**). (**D**) Detail of (**C**), arrowhead, showing normal retinal layers and RPE morphology. Subretinal ectopic nuclei, yellow arrowheads. Scale bar, 25 µm, also applies to panel (**B**). (**E**–**L**) Serial analysis of a retinal fold and neovascular lesions corresponding to **C**, red asterisk. Every fourth section obtained by sectioning is shown, corresponding to a spacing of 20 µm between the sections shown. Scale bar in (**L**), 100 µm, applies to panels (**E**–**L**). (**F′**,**H′**,**J′**,**L′)** Detail of panels (**F**,**H**,**J**,**L**). RPE layer doubling, yellow asterisks. Independent neovascular structures through the ONL (**H′**,**L′**, red arrowheads) are part of the same lesion. (**H′**) Scale bar, 25 µm, applies to panels (**F′**,**H′**,**J′**,**L′)**.

To characterize the *tvrm77* disease phenotype at the tissue and cellular level, mutant and B6 mice were examined at 2 months of age by histology. Retinal layers were well-ordered in the posterior eyes of B6 samples (Figure 5A,B). By contrast, the posterior eyes of *tvrm77* mice, which were documented by indirect ophthalmoscopy or fundus imaging to contain white-line lesions, included one or more prominent retinal lesions with altered lamination (Figure 5C,D, asterisks). Additionally, ectopic nuclei were noted in the subretinal space of *tvrm77* mice, which may correspond to immune cells mobilized to the retina–RPE interface, as observed in other models of retinal dystrophy [59,60,61,62].

Analysis of serial sections supported the features of white-line lesions as identified by OCT. At the start of the lesion, a small infolding of the ONL was observed above a perturbed RPE region, which included ectopic subretinal nuclei (Figure 5E–G). A more detailed view indicated a focal doubling of the RPE layer and voids in the subretinal space, which may correspond to excess subretinal fluid at this location (Figure 5F′). In a nearby section, an apparent neovascular structure was observed adjacent to a prominent outer retinal fold (Figure 5H,H′). Progressive distortion of the OPL and INL and a focal increase in total retinal thickness were observed as the folds became more pronounced. Subsequent sections exhibited a pseudorosette structure consisting of a thinned layer of photoreceptor nuclei surrounding an eosinophilic region (Figure 5I–L), which was contiguous with the eosinophilic inner and outer segments of photoreceptor cells observed in folds nearer to the lesion start (Figure 5H). The interior of the pseudorosettes included nucleated cells, which may be immune-related, and voids that may correspond to accumulated subretinal fluid. The presence of the pseudorosette structure in 16 consecutive sections suggests that it corresponds to the hyperreflective core and hyporeflective surrounding of white-line lesions observed by OCT. An additional neovascular structure was observed near the end of the collected series of sections (Figure 5L,L′). Taken together, these findings support the interpretation of the white-line lesions as retinal folds and confirm the close association of these lesions with one or more neovascular structures.

### 2.8. Fluorescence and Electron Microscopy of Retinal Folds, Detachment, and Neovascularization

**Figure 6:**
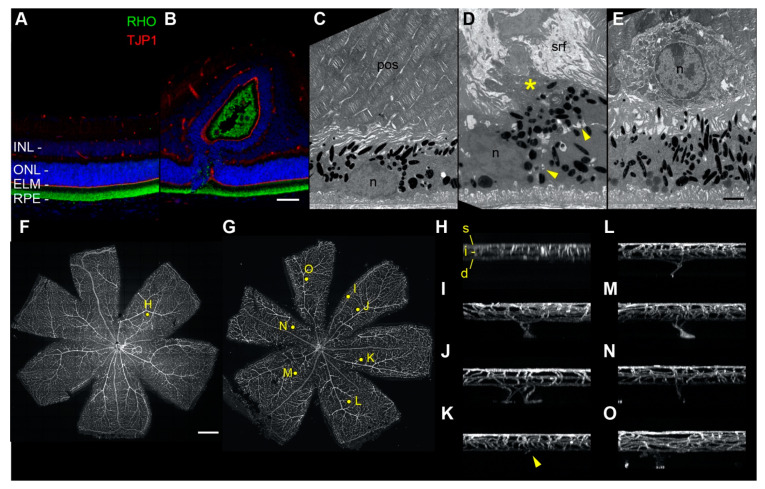
Fluorescence and electron microscopy analysis of control and *tvrm77* mice at 1 month of age. (**A**,**B**) Fluorescence microscopy of (**A**) B6 (n = 4) and (**B**) *tvrm77* (n = 5) mice with antibodies against rhodopsin (RHO) and ELM protein TJP1. Scale bar in **B**, 50 µm, applies to (**A**,**B**). (**C**–**E**) TEM of B6 (n = 4) and *tvrm77* (n = 5) posterior eyecups. Representative TEM of B6 samples (**C**) reveals normal apposition of photoreceptor outer segments (pos) with RPE cells. Nuclei, n. In *tvrm77* eyecups (**D**,**E**), subretinal fluid (srf) and debris (asterisk) accumulate above a region of RPE perturbation. A cell boundary (yellow arrowheads) can be seen separating the primary RPE layer from a displaced RPE cell or cellular process. In (**E**), a nucleated cell, likely an immune cell, lies at the retina–RPE interface. Scale bar in I, 2 µm, applies to (**C**,**D**)**.** (**F**,**G**) Maximum intensity projections of retinal flatmounts (n = 6) of B6 (**F**) or *tvrm77* (**G**) mice stained with isolectin B4. Yellow dots indicate regions selected for orthogonal display in panels (**H**–**O**). Scale bar in (**F**), 0.5 mm, applies to (**G**). (**H**) Orthogonal maximum intensity projection of a 0.3 mm square region of interest in panel (**F**). Normal superficial (s), intermediate (i), and deep (d) vascular beds appear in B6 retinas. (**I**–**O**) Projections of all detected neovascular lesions from the *tvrm77* retina in **G** are shown. The vessels appear to originate from the deep vascular bed and migrate toward the outer retina. In (**K**), the neovascular structure (arrowhead) is smaller compared to the others observed and may represent an early stage of lesion formation.

To detect additional features of *tvrm77* lesions, we examined fixed ocular tissues by fluorescence and transmission electron microscopy (TEM). Immunofluorescence microscopy of control B6 mice using antibodies against rhodopsin (RHO) revealed photoreceptor outer segments contacting the RPE (Figure 6A, green). Antibodies against tight-junction protein 1 (TJP1) revealed an intact ELM at the outer ONL boundary (Figure 6A, red). An identical arrangement was observed in unaffected regions of *tvrm77* eyes (Figure 6B). However, at *tvrm77* lesions, RHO staining was limited to the pseudorosette interior, and an intact TJP1 band encircling the interior was observed at the ONL boundary. The preservation of outer segments and ELM suggests that the lesions are created by the infolding of a developmentally intact retina. TEM of B6 eyecups indicated a normal apposition of photoreceptor outer segment with the RPE, which was disrupted in *tvrm77* mice by subretinal fluid and debris (Figure 6C,D). Occasional immune cells were observed at the retina–RPE interface, supporting the histological observations (Figure 6E). Flatmounts of B6 retinas stained with isolectin B_4_ to visualize blood vessels revealed three vascular layers with no evidence of abnormal vascular structures (Figure 6F,H). By contrast, *tvrm77* retinas exhibited neovascular structures departing from the deep vascular bed and extending toward the outer retina (Figure 6G,I–O). These results provide further evidence that retinal neovascular structures form in *tvrm77* mice.

### 2.9. Aberrant ERG Response

**Figure 7:**
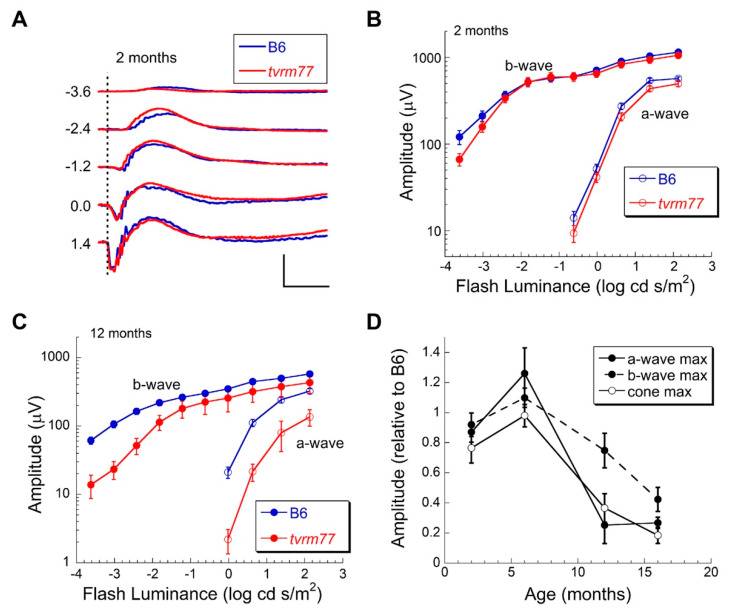
Strobe flash ERG analysis of B6 and *tvrm77* mice. (**A**) Representative ERGs obtained from 2-month-old B6 (blue) and *tvrm77* (red) mice exposed to strobe flash stimuli in log cd s m^−2^ presented to the dark-adapted eye. Calibration bars indicate 100 msec and 500 µV. (**B**) Luminance–response functions for the major components of the ERG obtained from 2-month-old mice. Data points indicate the mean (±SEM) response amplitude of six B6 and six *tvrm77* mice. (**C**) Luminance–response functions for the major components of the ERG obtained from 12-month-old mice. Data points indicate the mean (±SEM) response amplitude of five B6 and five *tvrm77* mice. (**D**) Response amplitudes of *tvrm77* mice plotted relative to B6 controls as a function of age. Responses of *tvrm77* mice were comparable to those of B6 mice up to 6 months of age and declined at older ages. Data points indicate mean (±) SEM of 3–15 mice.

**Figure 8:**
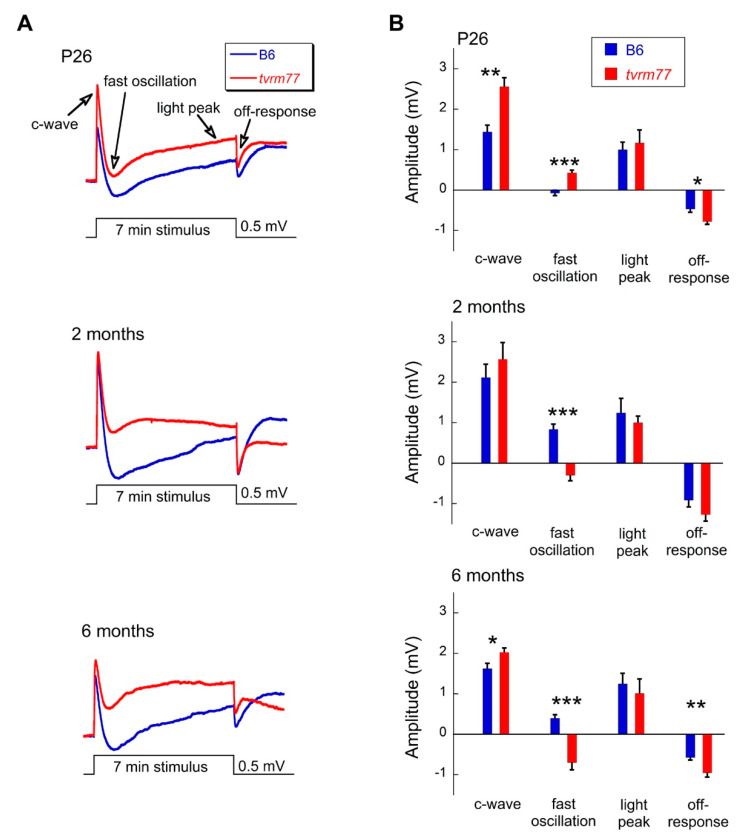
Analysis of the response properties of the RPE. (**A**) dc-ERG waveforms obtained in response to 7 min duration stimuli of 2.4 log cd m^−2^ from B6 and *tvrm77* mice at 26 days (P26), 2 months, or 6 months of age. Responses of *tvrm77* mice have a consistent change where the fast oscillation component remains above the pre-stimulus baseline. Waveforms indicate the average response of 4–10 mice. (**B**) Amplitude of the major response components of the dc-ERG. Bars indicate the mean (±SEM) response of 4–10 mice and asterisks indicate statistical significance: * *p* < 0.05 **; *p* < 0.01; *** *p* < 0.001.

To assess functional changes, ERGs were examined at multiple ages. Strobe flash ERGs were used to examine the response properties of the outer neural retina. At 2 months of age, ERGs of B6 and *tvrm77* mice were comparable under dark-adapted conditions (Figure 7A,B,D). These results indicate that the function of rod and cone photoreceptors as well as of bipolar cells, which generate the major components of the strobe flash ERG, is maintained in *tvrm77* animals. At 12 months of age, ERGs of *tvrm77* mice were significantly reduced (Figure 7C). This reflects an age-dependent decline in response amplitude that began after 6 months of age (Figure 7D). RPE function was examined using dc-ERG recordings. Because the dc-ERG is generated secondary to rod photoreceptor activity [63] and is thus impacted by photoreceptor degeneration [64], we focused on mice at 6 months of age or younger, in which the rod-driven ERG a-wave remained comparable to that of B6 mice (Figure 7D). At every age examined (26 days, 2 months, and 6 months of age), the *tvrm77* waveform was dramatically different in comparison to B6 (Figure 8A).

While the c-wave and the off-response were comparable or larger in *tvrm77* as compared to B6 mice, the fast oscillation (FO) and light peak (LP) components were reduced in the mutant animals (Figure 8B).

## 3. Discussion

In this report, we characterize a new mutant allele of the mouse sodium bicarbonate cotransporter gene *Slc4a5* identified by the TVRM program [12,13]. Our results support the hypothesis that disease phenotypes in *tvrm77* mice are secondary to RPE dysfunction caused by a loss of SLC4A5 function. As discussed below, this conclusion is based on evidence of early abnormalities in mutant mice, including RPE-specific dc-ERG responses and subretinal fluid accumulation accompanied by retinal neovascularization and outer retinal folds. Our findings suggest a role for SLC4A5 relevant to the function of the outer retinal BRB, perhaps through its function in electrolyte and fluid transport across the RPE.

### 3.1. RPE Expression of Slc4a5 and Its Disruption in tvrm77 Mice

Previous studies of three *Slc4a5* mouse knockout models indicated functional expression of the gene and SLC4A5 protein in epithelial cells that regulate sodium bicarbonate transport at important interfaces between the circulation and tissue compartments, including the blood–CNS barrier of the choroid plexus [21,23] and the collecting ducts and tubules of the kidney [26,64,65]. Previous analysis of ocular pathology in one of the knockout strains revealed retinal detachment, retinal ganglion cell and photoreceptor cell loss, outer segment shortening and dysmorphology, and decreased ERG response amplitudes [21]. Immunohistochemistry with a non-commercial SLC4A5 antibody revealed prominent staining in the OPL, which was absent in the knockout strain. These findings led the authors to suggest that SLC4A5 might be important for pH buffering at the photoreceptor synaptic terminus, possibly in a light-dependent fashion. A potential contribution of the RPE to the ocular phenotypes was not discussed.

We describe a new *Slc4a5* allele in *tvrm77* mice that disrupts *Slc4a5* splicing and causes a 19.7-fold decrease in *Slc4a5* mRNA levels. Multiple alternate transcripts were confirmed in B6 control mice, consistent with transcription from two promoters, and were altered similarly in the *tvrm77* model. The *tvrm77* transcripts were predicted to encode SLC4A5 polypeptides that were prematurely truncated near the amino terminus and lacked the transmembrane domain required for transport function. Thus, the *Slc4a5^tvrm77^* allele is likely to be a null allele, at least in the eye. Database and literature reports identify *Slc4a5* mRNA in the RPE, and our immunohistochemistry results confirmed that SLC4A5 is produced by cultured human RPE cells. Based on these results and the evidence for SLC4A5 presence and function in epithelial cells, we suggest that the ocular phenotypes observed in *tvrm77* mice, as well as in the knockout strain studied previously [21], are attributable at least in part to a loss of SLC4A5 in the RPE.

### 3.2. Early RPE-Specific ERG Defects

Our ERG studies identified abnormalities in two ERG components generated by the RPE, the FO and LP. Both components are generated by the basal RPE membrane. The major factor underlying the FO is a hyperpolarization of the basal membrane of the RPE in response to the initial decline in subretinal [K^+^] [65,66]. The LP reflects a depolarization of the basal membrane of the RPE [67]. Although the specific ion channel that underlies this ERG component has not been determined, it is interesting to note that heterozygous *Clcn2^nmf240^* mice expressing a single functional allele have a selective reduction of LP amplitude [68]. In *tvrm77* mice, only the FO and LP were reduced in amplitude. This suggests that the primary impact of losing SLC4A5 function is on the basal membrane where these components are generated. In comparison, the c-wave and off-response components remained normal or were actually larger in amplitude in *tvrm77* as compared to B6 mice. This amplitude increase could reflect how the underlying components interact to define the final waveform. In support of this idea, larger amplitude c-waves were obtained from mice expressing only a single allele of *Kir4.1* compared to B6 animals, reflecting the contribution of Kir4.1 conductance to the negative-polarity slow PIII component, which combines with the positive-polarity component generated by the apical RPE membrane to define the c-wave [69].

### 3.3. Role of RPE SLC4A5 in Fluid Transport across the Outer BRB

Physiological studies of frog, bovine, human, and cultured human RPE have identified a system of integral membrane transporters and intracellular enzymes responsible for net movement of HCO_3_^−^ and H_2_O from the subretinal space to the choroidal circulation [7,8,9,70,71]. This system is postulated to remove excess CO_2_ and H_2_O generated by the high metabolic activity of photoreceptor cells, maintaining a negative hydrostatic pressure that ensures tight adhesion of the retina to the RPE apical surface. The functional importance of an electrogenic basolateral sodium bicarbonate cotransporter to this system was established by physiological studies [70] and further supported by mathematical modeling [9]. SLC4A4 (NBC1) and SLC4A5 were considered as candidates for this transporter based on high levels of the corresponding transcripts in native and cultured human RPE [70,72]. SLC4A5 was the preferred candidate based on a comparison of HCO_3_^−^ transport mechanisms in the RPE and choroid plexus epithelium (CPE), which is developmentally related to the RPE [73,74]. The Na^+^:HCO_3_^−^ 1:3 stoichiometry of SLC4A5-mediated efflux in the CPE [20] matched the inferred efflux stoichiometry of the unidentified electrogenic sodium bicarbonate transporter in the basolateral RPE [70]. Furthermore, the CPE transports sodium bicarbonate and fluid from the circulation to the cerebrospinal fluid, while the RPE moves these substances in the opposite direction, from the subretinal space to the circulation. Thus, the localization of sodium bicarbonate transporters SLC4A4 and SLC4A8 in the CPE basolateral membrane [73,74] and SLC4A5 in the apical membrane [20] is expected to be reversed in the RPE [70]. SLC4A4 is found at the apical membrane of RPE cells [75], reinforcing this prediction. A reversed apicobasal distribution of SLC4A5 and other sodium bicarbonate transporters in the RPE is not unexpected, because the distribution of other RPE membrane transporters, such as the monocarboxylate transporter SLC16A1, the Na^+^,K^+^-ATPase, and the cystic fibrosis transmembrane conductance regulator, is opposite to their distribution in the CPE and other epithelia [71]. Thus, existing evidence predicts that SLC4A5 is an electrogenic sodium bicarbonate transporter in the basolateral membrane of the RPE.

In support of this prediction, we detected SLC4A5 in human iPSC-derived RPE cells. However, most of the protein was localized to the Golgi apparatus, as reported in renal proximal tubule cells [76]. Thus, we cannot exclude the possibility that SLC4A5 functions predominantly in the Golgi apparatus, possibly controlling the cellular trafficking and/or localization of other solute carriers that determine RPE electrolyte and fluid transport. Nevertheless, SLC4A5 was also detected in puncta at the basolateral membrane, and our ERG findings indicated a transport defect at the basolateral membrane in *tvrm77* mice. In addition, we found evidence for subretinal fluid accumulation based on the hyporeflective OCT band in the subretinal space of *tvrm77* mice at about 1 month of age that became more pronounced in older animals, indicating a disruption of fluid transport from the subretinal space. Together with the earlier physiological studies of RPE transport, these findings raise the possibility that basolateral SLC4A5 regulates sodium bicarbonate and water efflux across the RPE and outer BRB. Future studies to compare the electrolyte and fluid transport properties of B6 and *tvrm77* RPE may test this hypothesis directly.

### 3.4. A Possible Model of Exudative Retinal Detachment and Neovascularization Type 3

As the goal of the TVRM program is to provide mouse models of human ocular disease [12,13,14], we searched the clinical literature for reports of disease phenotypes resembling those of *tvrm77* mice. Retinal detachments are classified as exudative (serous), due to subretinal fluid accumulation; rhegmatogenous, due to retinal breaks that allow the vitreous to infiltrate below the retina; and tractional, due to the contraction of scar tissue on the vitreal surface of the retina [77]. Our OCT and histological analysis did not indicate retinal breaks or scar tissue on the vitreal surface of the retina, suggesting that *tvrm77* mice model exudative retinal detachment.

Retinal neovascular lesions have been reported in several ocular conditions. Idiopathic retinal telangiectasia with exudation [78], a spectrum of unilateral, sporadic disease phenotypes that includes forms of Coats disease, is characterized by capillary networks, which emanate from the deep retinal plexus, and exudative retinal detachment. A form of age-related macular degeneration, macular neovascularization Type 3 (MNV3), also called retinal angiomatous proliferation (RAP), consists of neovessels that originate from the deep retinal plexus and in late stages anastomose with the choroidal circulation [79,80]. Macular telangiectasia type 2 [81] is a bilateral neovascular disease presenting simultaneously with local retinal detachment in the form of subfoveal fluid accumulation in 1–2% of affected patients [82,83]. These diseases are associated with photoreceptor loss [78,79,80,81]. Thus, *tvrm77* mice may recapitulate aspects of retinal neovascularization and degeneration as observed in these diseases.

### 3.5. A Possible Model to Study Retinal Fold Formation

Although retinal folds in humans are not known to have genetic associations, several types of folds have been observed in rhegmatogenous retinal detachment (RDD) [84] and, more frequently, as a postoperative complication of surgery to correct this condition [49,51,84,85,86,87,88]. Hydration folds, as detected by OCT prior to surgery, feature a corrugated appearance of the outer retina, which is typically completely detached from the underlying RPE [51,84]. By contrast, postoperative outer retinal folds are less frequent and appear as hyperreflective retinal intrusions between the RPE and outer plexiform layers. These lesions are observed in reattached retinas, although they are often accompanied by local detachment at the site of the fold. Full-thickness folds compromise the entire retina and may include a central hyperreflective intrusion. Like outer retinal folds, full-thickness folds are also accompanied by local detachment. Hydration, outer retinal, and full-thickness folds have been proposed to arise from an excess of subretinal fluid, which temporarily overwhelms the fluid extrusion capabilities of the RPE. With time, outer retinal folds are typically resolved without further intervention, presumably due to effective fluid extrusion by the RPE. The clinical appearance of outer retinal and full-thickness folds by fundus imaging and OCT is similar to that of white-line lesions observed in *tvrm77* mice. By analogy to outer retinal and full-thickness folds in humans, the lesions in *tvrm77* mice may fail to resolve due to a genetic defect in fluid extrusion from the subretinal space. Further studies of *tvrm77* mice may reveal whether these lesions are a consequence of exudative retinal detachment caused by the *Slc4a5* mutation or an independent phenomenon and may yield insights into the post-surgery management of outer retinal and full-thickness folds.

The occurrence of retinal folds in dogs, also called canine retinal dysplasia, has been observed in non-syndromic (sporadic) multifocal retinal dysplasia and in syndromic multifocal retinal dysplasia among Labrador retrievers and Samoyeds harboring the *drd1* and *drd2* (dwarfism with retinal dysplasia 1 and 2) mutant alleles [52]. Strikingly, retinal detachment accompanies retinal folds in severe cases of the syndromic disease [52], as in *tvrm77* mice. The cross-sectional appearance of lesions observed in affected dogs by OCT included hyperreflective cores in the outer retina that distort the overlying inner nuclear layer and cause a local retinal bulge, identical to the white-line lesions observed in *tvrm77* mice. Retinal folds in affected *drd1* and *drd2* dogs are found near superior venules and arterioles at the posterior pole, suggesting a possible retinal vascular contribution to fold formation. Although the *drd1* and *drd2* loci harbor mutations in the *COL9A3* and *COL9A2* genes, respectively [89], these mutations did not segregate with retinal folds, so the causative gene or genes for this phenotype remain to be identified. It would be of interest to examine whether canine *SLC4A5* or other known regulators of RPE fluid flow are altered in *drd1* and *drd2* canines exhibiting retinal dysplasia.

A previous study of *Slc4a5* knockout mice, in which ocular phenotypes were characterized, did not report neovascular lesions or retinal folds [21]. The study included OCT and microscopy of fixed ocular sections, which might have detected folds if they had occurred within the region analyzed. However, in the absence of a technique to survey the entire posterior eye, such as indirect ophthalmoscopy, it is possible that such lesions occurred but were not detected in the knockout strain. Alternatively, differences in the *Slc4a5* allele (splice mutation versus gene trap) or in strain genetic background may have resulted in an altered phenotype. In the previous study, the mutation was engineered in 129S5/SvEvBrd embryonic stem cells, which were implanted into B6 blastocysts and subsequently maintained in this strain. The mixed genetic background of the resulting *Slc4a5* knockout mice may have resulted in a decreased prevalence of neovascular lesions and retinal folds. Ocular phenotypes for two other *Slc4a5* knockout strains [23,26] have not yet been reported.

### 3.6. Study Limitations and Recommendations for Future Work

There are several limitations to this study. We were unable to detect mouse SLC4A5 in native tissue using commercially available antibodies and therefore studied polarized human iPSC-derived RPE cells in transwell culture. Antibodies that detect SLC4A5 in the native mouse RPE are needed to determine basolateral localization in vivo. Our studies did not address the possible contribution of *Slc4a5* expression in other retinal cell types to *tvrm77* phenotypes. For example, the expression of *Slc4a5* mRNA has been documented in horizontal cells [35], and these cells have been implicated in retinal vascular development [90]. Cell-specific *Slc4a5* ablation [91] may distinguish whether a loss of SLC4A5 function in these cells or the RPE is sufficient to cause retinal detachment, folds, neovascularization, and degeneration. Although we have backcrossed *tvrm77* with B6 mice for six generations, we cannot exclude the possibility that linked ENU-induced mutations in additional genes may influence the observed phenotypes. Further backcrossing may reduce this possibility. Finally, our studies do not resolve disease progression sufficiently to establish the order of pathological events that cause the observed phenotypes. Longitudinal microscopy studies that include early postnatal time points may provide clues to the cause of subretinal fluid accumulation and reveal the relationship between retinal neovascularization and the formation of retinal folds.

### 3.7. Summary

Our studies of a new *Slc4a5* mutant allele, *tvrm77*, in mice support a role for the SLC4A5 protein in RPE cells at the outer BRB. The mutant mice may serve as a model of exudative retinal detachment, retinal neovascularization, and retinal dysplasia. Although we focused exclusively on the ocular phenotype, we note that previously reported *Slc4a5* mutant strains exhibited abnormalities in the epithelia of the bile duct, liver, brain, and kidney [17,18,19,20,21,22,23]. Thus, the *tvrm77* mouse may be of value in defining the role of SLC4A5 in the eye and also in these other organs.

## 4. Materials and Methods

### 4.1. Mice, Mutagenesis and Mapping

C57BL/6J (B6; JAX stock #000664) mice were obtained from the production colony at JAX (Bar Harbor, ME, USA), mutagenized with ENU, and screened for ocular defects by the TVRM program, yielding the *tvrm77* line as described [12,13]. The line was initially maintained by backcrossing to B6 for five generations (N_5_) with intercrossing at each generation to identify affected mice; once the mutation was identified, an additional backcross was conducted, and N_6_ mice were maintained by intercrossing. The full designation for this model is C57BL/6J*^-^Slc4a5^tvrm77^*/Pjn; for simplicity, we retain the original strain designation *tvrm77*, and unless otherwise indicated, the genotype is homozygous for the mutant allele. Mice were housed in the Research Animal Facility at JAX on a 12 h light/12 h dark cycle and provided an NIH31 (6% fat) diet and acidified water ad libitum. All animal procedures were approved by the Institutional Animal Care and Use Committees of the JAX or Cleveland Clinic and adhered to the ARVO Statement for the Use of Animals in Ophthalmic and Vision Research.

To map the chromosomal location of the *tvrm77* mutation, affected females (presumed homozygotes) were mated to male DBA/2J (D2; JAX stock #000671) mice to generate F_1_ progeny which were subsequently intercrossed. F_2_ progeny were assessed by indirect ophthalmoscopy at 8–10 weeks of age, and DNA was isolated from tail snips using a modified NaOH lysis protocol [92]. For the genome scan, DNA pools of 10 affected and 10 unaffected mice were genotyped using a panel of 99 simple sequence length polymorphic markers (SSLP) [93] that span the genome (The Jackson Laboratory Fine Mapping Facility, Bar Harbor, ME, USA). The disease gene was localized to a 30 Mb region on mouse Chr 6 between SSLP markers *D16Mit16* and *D6Mit104*. To further refine the critical region, a second mapping cross was generated by intercrossing F_1_ progeny from an outcross of homozygous B6-*tvrm77* to C3A.BLiA-*Pde6b*^+^/J (JAX stock #001912, also known as “sighted C3H”). In total, 751 F_2_ DNAs (375 from B6 × D2 and 376 from B6 × C3A.BLiA-*Pde6b*^+^/J intercrosses) were tested for recombination with *D6Mit188* and *D6Mit9*, markers flanking the critical region (Chr 6:75447583–87426034 bp). The affected status of critical recombinants was confirmed by progeny tests.

### 4.2. Mutation Analysis

To determine the causative gene mutated in *tvrm77*, total RNA was prepared from whole eyes of *tvrm77* and B6 control mice using TRIzol reagent (Life Technologies, Carlsbad, CA, USA) according to the manufacturer’s instructions, and cDNA was generated with a Retroscript reverse transcription kit (Ambion, Inc., Austin, TX, USA). The coding region of the candidate gene *Slc4a5* was amplified from cDNA using PCR, and purified products were sequenced. Subsequently, DNA from affected and unaffected F2 progeny and parental strains including B6, D2, and C3A.BLiA-*Pde6b*^+^/J was sequenced and compared to determine the genomic position of the *tvrm77* mutation in *Slc4a5*.

### 4.3. Analysis of Slc4a5 RPE Transcripts

RPE was isolated from 1-month-old *tvrm77* mutant (n = 3) and B6J control (n = 4) eyes as described [94]. Briefly, eyes were enucleated immediately from euthanized mice and placed into phosphate-buffered saline (PBS). Angled Vannas scissors were used to remove the anterior segment and lens just below the limbus. The retina was carefully removed, exposing the RPE. Each eyecup was incubated for 10 min in RNAprotect Cell Reagent (Qiagen, Hilden, Germany), with gentle agitation every few minutes. The posterior eyecup was removed, and the RPE cell suspension was centrifuged at 3000× *g* for 5 min. Supernatant was removed, and the cell pellet was flash frozen in liquid nitrogen and stored at −80 °C. RNA was extracted from the cell pellet using RNeasy Micro Kit (Qiagen, Hilden, Germany) following manufacturer’s recommendations. cDNA was generated using 350 ng total extracted RNA using RETROscript Reverse Transcription (Ambion, Inc., Austin, TX, USA) according to manufacturer’s recommendations.

For qPCR, reactions were run in duplicate on an iCycler (Bio-Rad, Hercules, CA, USA) at 95 °C for 30 s, followed by 40 cycles of 95 °C for 4 s and 60 °C for 20 s using iTaq Universal SYBR Green Supermix (Bio-Rad, Hercules, CA, USA). Each reaction contained 10 ng cDNA, 200 nM of each primer, and 10 μL iTaq Universal SYBR Green Supermix in 20 μL total volume. The fold changes in expression were calculated using 2^−ΔΔCt^ using *Actb* as a reference gene. Primer sequences for *Slc4a5* include RT-F, GACCAGCCATTCATTGCATTT and RT-R, CTCTCCCGGAAGGTCCTAATA; those for *Actb* include F1, CCAGTTCGCCATGGA-TGACGATAT and R1, GTCAGGATACCTCTCTTGCTCTG.

To determine the effects of *Slc4a5* mutation on mRNA splicing, RPE cDNA was amplified using primers specific for *Slc4a5* exons: mF, CCCCTTTCTTTCTCATTTGC; mR, TTTTCCGCTGGTCTGTGTT; and F03, TTTGGAGCCTGGTGTCCAC. For samples amplified using the F03 primer, a second primer, bF CCTGAAACCAGGCAAGCCTC, was used in a subsequent amplification step by nested PCR.

### 4.4. Human Pluripotent Stem Cell Culture

WA09 hESC (WiCell) and A04 hiPSC, a clonal derivative of HPSI0114i-kolf_2 [95], were provided by Dr. Bill Skarnes, JAX. Cells were maintained in StemFlex defined culture medium (Thermo Fisher Scientific, Waltham, MA, USA) as adherent cultures on a substrate of a recombinant fragment of human vitronectin (Synthemax, Corning, Corning, NY, USA). Cells were routinely passaged with a non-enzymatic dissociation reagent (ReLeSR, Stem Cell Technologies, Vancouver, CB, Canada). Cells were used within 15 passages from diploid master stocks, and undifferentiated cultures expressed cell surface markers and transcription factors characteristic of the pluripotent state. Bimonthly testing confirmed the absence of contamination by mycoplasma or other adventitious microbial agents. Human embryonic stem cell research was approved by the University of Connecticut Stem Cell Research Oversight Committee on behalf of JAX.

### 4.5. Human RPE Cell Differentiation and SLC4A5 Expression

hESC or hiPSC differentiation into RPE followed the multistage protocol (differentiation, enrichment, and maturation phases) as described [40,41]. Undifferentiated hESC or hiPSC grown in defined medium (see above) were transferred to Matrigel-coated plates and incubated in basal medium (DMEM:F12 with N2B27 and non-essential amino acids) supplemented with the following factors in this sequence: nicotinamide, noggin, DKK-1, and IGF1 from Day 0–2; addition of FGF2 to the Day 0–2 combination from Day 2–4; Activin A, DKK-1, and IGF-1 from Day 4–6; Activin A and SU5402 from Day 6–8; Activin A, SU5402, and CHIR99021 from Day 8–14. Following induction of differentiation, cells were harvested and transferred to X-VIVO-15 medium on Matrigel-treated plates from Day 14–42 and then harvested and cultured in the same medium and substrate on tissue culture flasks, slides, or transwell inserts. Cells were fixed or harvested for immunoblot analysis between Day 30 and Day 120. Differentiation to RPE was confirmed by immunostaining with antibodies to PEDF, ZO1, TIMP3, BEST1, RPE65, and transmission electron microscopy to demonstrate melanosome granules.

Cultures on multiwell slides or transwell inserts were fixed for 30 min in 4.0% paraformaldehyde in phosphate-buffered saline at 4 °C. Antibodies used for immunostaining were rabbit anti-human SLC4A5 polyclonal (HPA036621; Sigma, St. Louis, MO, USA), mouse anti-GORASP2 (CL2610; Abcam, Cambridge, UK), and mouse anti-BEST1 (E6-6; Abcam). Secondary antibodies were Alexa488 goat anti-mouse Ig or Alexa594 goat anti-rabbit (both from Thermo Fisher Scientific, Waltham, MA, USA). Nuclei were counterstained with 4′,6-diamidino-2-phenylindole. Stained preparations were examined using either a Leica DMi8 inverted microscope (Leica, Wetzlar, Germany) equipped with epifluorescence illumination or a LSM800 confocal microscope (Carl Zeiss, Oberkochen, Germany). Images were processed using the respective manufacturer’s software.

### 4.6. Live Imaging of Mouse Eyes

Noninvasive fundus imaging with a Micron III or IV retinal camera (Phoenix Research Laboratories, Pleasanton, CA, USA) and a spectral domain OCT Envisu R2210 imaging system (Leica, Wetzlar, Germany) were performed as described [96]. Briefly, Micron camera videos of 100 frames were registered, averaged, digitally sharpened, and adjusted for brightness and contrast in Fiji/ImageJ with a custom macro (available through JAX Eye at the ImageJ update site). OCT image volumes were registered and averaged from 5 or 10 replicate rectangular scans (1000 × 100 × 1). Fundus images were rotated in Fiji/ImageJ to match *en face* OCT stacks based on retinal vascular landmarks. OCT layer thicknesses were determined from a B-scan selected from the OCT volume and centered on the optic nerve head. Segmented lines roughly 200 µm in length were drawn along the ELM on both sides of the optic nerve head roughly 400 µm from its center, fit to a spline curve, interpolated (20 pixels), and straightened. A full-width vertical line was centered along the straightened images, and the Plot Profile tool was applied to generate a plot of grayscale intensity versus axial position. Plot coordinates were imported into a custom Excel template that determined the distance between layers as measured at the midpoints between the peak and valley of grayscale intensity, which define the boundaries of each retinal layer. Values from each side of the optic nerve head were averaged.

### 4.7. Mouse Histology and Immunohistochemistry

Mice were sacrificed by CO_2_ asphyxiation. Enucleated eyes were placed in methanol:acetic acid:PBS (3:1:4) and fixed overnight at 4 °C. The following day, tissues were dehydrated in an alcohol series and embedded in paraffin. Tissues were cut into 5 µM sections and mounted on Colorfrost plus (Thermo Fisher Scientific) glass slides. For histological analysis, sections were stained with hematoxylin and eosin and visualized by light microscopy. For immunohistochemistry (IHC), retinal sections were de-paraffinized and blocked in 1× PBS, 0.5% Triton, and 2% normal donkey serum for 30 min at room temperature. Tissues were then incubated in mouse anti-rhodopsin (MilliporeSigma, Burlington, MA, USA; MA5-11741) and rabbit anti-TJP1 (Thermo Fisher Scientific, Waltham, MA, USA; 61–7300) antibodies overnight at 4 °C. After several washes in 1× PBS, sections were incubated with a Cy3-conjugated (Jackson Immunoresearch, West Grove, PA, USA) rabbit and Alexa Fluor 488-conjugated (Life Technologies, Carlsbad, CA, USA) mouse anti-IgG secondary antibody (1:200) and stained with DAPI (Invitrogen, Waltham, MA, USA; D1306) to detect nuclei. Localization of rhodopsin and ezrin were visualized by fluorescent microscopy (DMLB, Leica), and images were acquired with QCapture software (QImaging, Surrey, BC, Canada).

### 4.8. Transmission Electron Microscopy

Enucleated eyes from *tvrm77* and C57BL/6J mice at 1 month of age were immersed in 2.5% *w/v* glutaraldehyde, 2% *w/v* paraformaldehyde solution, 0.1 M sodium phosphate, pH 7.2. The anterior segment was immediately removed, and the remaining posterior segment was incubated in fixative for 3–4 h at room temperature followed by storage in 10-fold-diluted fixative for at least 12 h at 4 °C. Eyecups were then transferred to 0.1 M phosphate buffer and cut into transverse blocks (1 × 2 mm^2^). Tissue specimens were post-fixed in 1% *w*/*v* osmium tetroxide, dehydrated, and embedded in Embed 812 resin. Ultrathin sections were stained in uranyl acetate and lead citrate and examined under a JEOL JEM-1230 transmission electron microscope (JEOL, Akishima, Tokyo, Japan).

### 4.9. Assessment of Retinal/RPE Adhesion

To determine the extent of retinal adhesion to the RPE, eyes were collected from B6 and *tvrm77* mice at 1 month of age after CO_2_ asphyxiation and dissected as described [32]. Enucleated eyes were dissected in 20 mM HEPES-buffered Hank’s saline solution containing calcium and magnesium (Mediatech, Manassas, VA, USA) at room temperature. A circumferential cut was made below the limbus, and the iris, cornea, and lens were removed. The posterior eyecup was placed in a clean petri dish and flattened with one radial cut toward the optic nerve. The retina was peeled from the RPE by gentle separation with forceps, snap frozen, and stored at −80 °C. To quantify adhesion, samples were sonicated in lysis buffer (1× NuPAGE LDS Sample Buffer NP0007 and 1× NuPAGE Sample Reducing Agent NP0004, Thermo Fisher Scientific, Waltham, MA, USA) and centrifuged at 1000 rpm for 1 min to pellet any remaining whole tissue. The supernatant was heated at 100 °C for 10 min prior to loading. A dilution series of the B6 samples was used to standardize the Western signal. Samples were run on a 10% Mini-Protean TGX Precast acrylamide gel (Bio-Rad, Hercules, CA, USA; 4561035) and transferred to nitrocellulose membranes. Membranes were probed with antibodies against ezrin (1:1000; Cell Signaling, 3145), chemiluminescence was detected using Clarity Western ECL Substrate (Bio-Rad, Hercules, CA, USA; 1705060), and total protein was assessed by Ponceau S staining. Membranes were imaged using an Azure c600 bioimaging system. The raw integrated densities of the ezrin bands and of Ponceau S bands on the same blots were determined using Fiji following subtraction of background intensity determined in empty lanes. Density values for the standard series were plotted against load volume and fit to a hyperbolic or sigmoidal curve in Prism, which was used to calculate the equivalent load volumes for all samples. Relative ezrin was determined by dividing the equivalent load volumes of the ezrin band by those of the corresponding total protein. Relative ezrin values were normalized by dividing by the mean value for all B6 samples.

### 4.10. Fluorescence Microscopy of Retinal Flatmounts

Retinal flatmounts were prepared as described. Briefly, eyes were removed and immediately fixed on ice in PBS containing 4% *w/v* paraformaldehyde. Eyecups were prepared within 30 min and cut radially before separating the retina from the RPE/choroid/sclera. Following overnight blocking at 4 °C with 5% normal donkey serum (Jackson ImmunoResearch, West Grove, PA, USA; 017-000-121), retinas were washed twice with PBS and stained with isolectin B_4_ AlexaFluor 647 conjugate (Thermo Fisher Scientific, Waltham, MA, USA; I32450) diluted 1:100 in PBS, 1 mM CaCl_2_, 0.5% Triton X-100. After staining for four days at 4 °C, samples were rinsed twice with PBS and mounted in VectaShield (Vector Laboratories, Burlingame, CA, USA). Tiled z-stacks (2.0 µm step size) capturing the full thickness of the entire retina were acquired at 20× by using a translational stage and ApoTome accessory of an Axio Observer Z1 microscope (Carl Zeiss, Oberkochen, Germany). Tiles were stitched with ZEN 2.6 software and exported in TIFF format to Fiji/ImageJ for maximum intensity projection and to adjust brightness and contrast. To display neovascular structures, full-thickness regions of interest, in which vascular deviations appeared below the deep vascular plexus, were selected from the image volume. A maximum intensity z-projection was generated from an orthogonal reslice of these regions of interest.

### 4.11. Electroretinography

ERG components generated by the outer retina and RPE were evaluated in two recording sessions using published protocols [97].

## Data Availability

Data are contained within the article or Supplementary material. Additional supporting data are openly available in Dryad at https://doi.org/10.5061/dryad.fttdz08vd.

## Supplementary Materials

The following are available online at https://www.mdpi.com/article/10.3390/ijms23042220/s1.
