# Supplementary figures and images for: A Splicing Mutation in Slc4a5 Results in Retinal Detachment and Retinal Pigment Epithelium Dysfunction
Source: Int J Mol Sci. 2022 Feb 17;23(4):2220. doi: 10.3390/ijms23042220 (PMC8875008; doi:10.3390/ijms23042220)

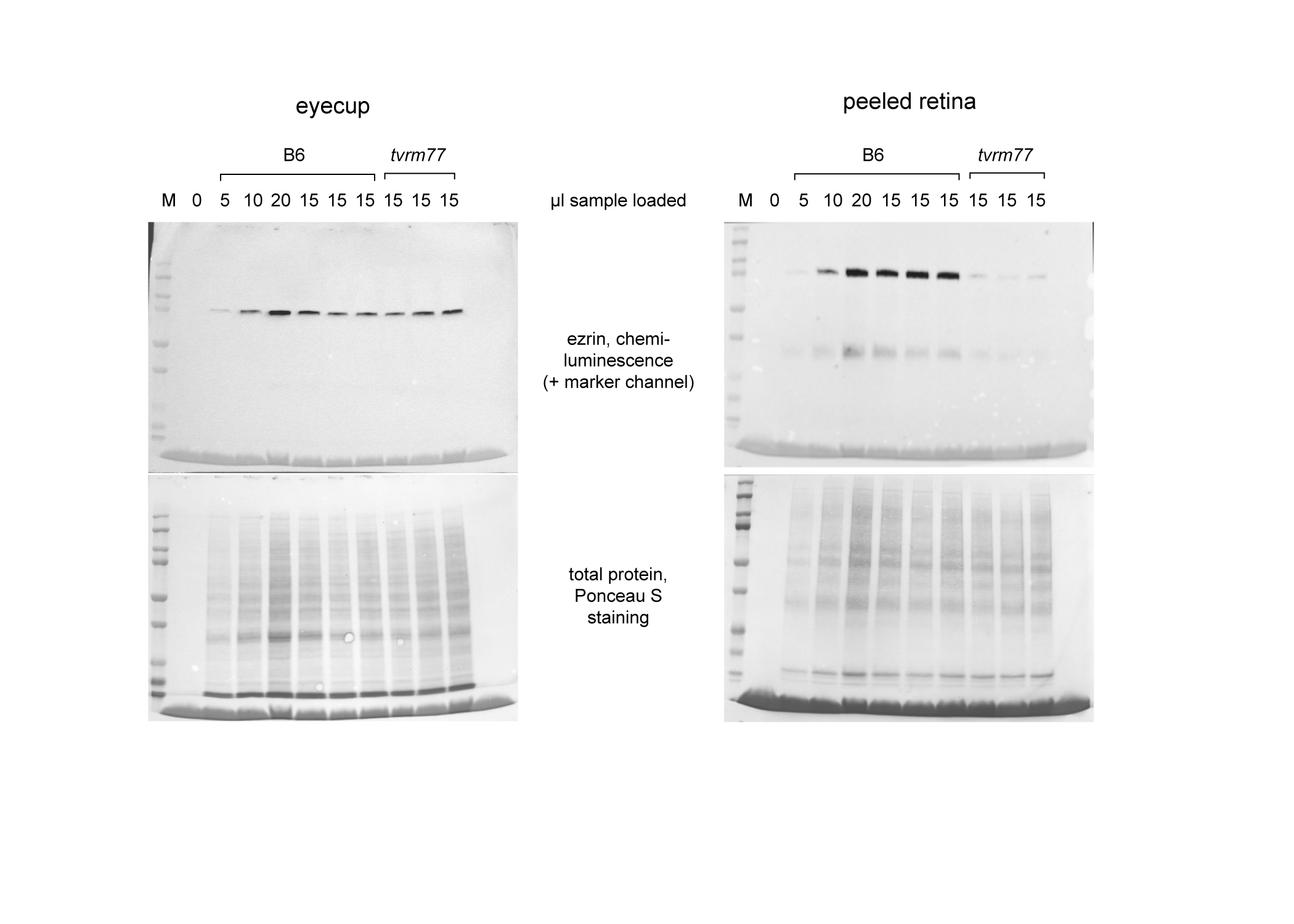

Supplement: Supplementary file 1 [file ijms-23-02220-s001.zip › Revised Submission Supplement/Figure S2.tiff]

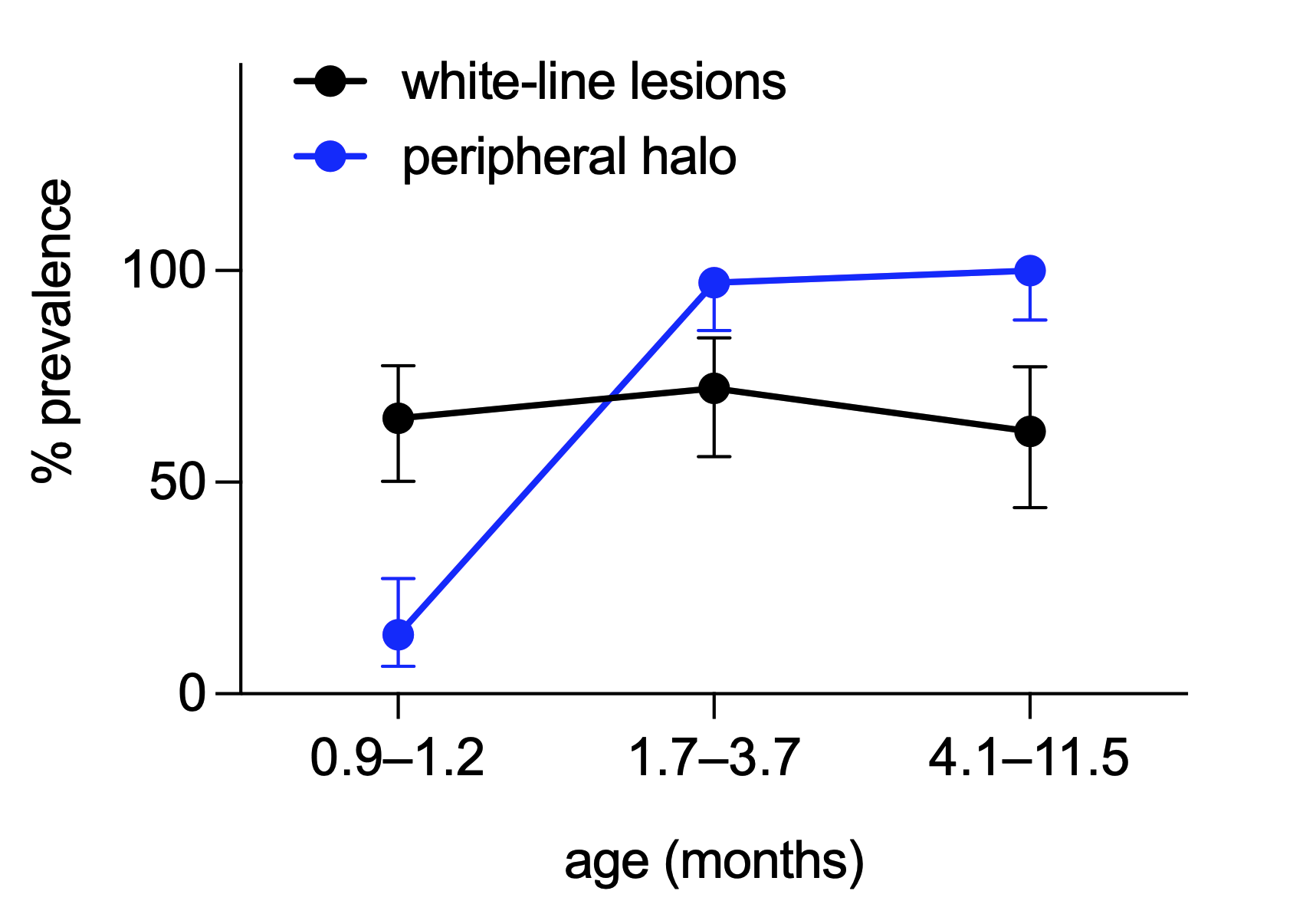

Supplement: Supplementary file 1 [file ijms-23-02220-s001.zip › Revised Submission Supplement/Figure S1.tiff]
